# Cooperation of regulatory RNA and the RNA degradosome in transcript surveillance

**DOI:** 10.1093/nar/gkae455

**Published:** 2024-06-06

**Authors:** Katarzyna J Bandyra, Kathrin S Fröhlich, Jörg Vogel, Marina Rodnina, Akanksha Goyal, Ben F Luisi

**Affiliations:** Department of Biochemistry, Sanger Building, University of Cambridge, Tennis Court Road, Cambridge CB2 1GA, UK; Department of Chemistry, Biological and Chemical Research Centre, University of Warsaw, Zwirki i Wigury 101, 02-089 Warsaw, Poland; Institute for Molecular Infection Biology, University of Würzburg, Josef-Schneider-Str. 2, 97080 Würzburg, Germany; Institute of Microbiology, Friedrich Schiller University Jena, 07743 Jena, Germany; Institute for Molecular Infection Biology, University of Würzburg, Josef-Schneider-Str. 2, 97080 Würzburg, Germany; Helmholtz Institute for RNA-based Infection Research (HIRI), Helmholtz Center for Infection Research (HZI), Josef-Schneider-Str. 2, 97080 Würzburg, Germany; Max Planck Institute for Multidisciplinary Sciences, Göttingen, Germany; Max Planck Institute for Multidisciplinary Sciences, Göttingen, Germany; Department of Biochemistry, Sanger Building, University of Cambridge, Tennis Court Road, Cambridge CB2 1GA, UK

## Abstract

The *ompD* transcript, encoding an outer membrane porin in *Salmonella*, harbors a controlling element in its coding region that base-pairs imperfectly with a ‘seed’ region of the small regulatory RNA (sRNA) MicC. When tagged with the sRNA, the *ompD* mRNA is cleaved downstream of the pairing site by the conserved endoribonuclease RNase E, leading to transcript destruction. We observe that the sRNA-induced cleavage site is accessible to RNase E *in vitro* upon recruitment of *ompD* into the 30S translation pre-initiation complex (PIC) in the presence of the degradosome components. Evaluation of substrate accessibility suggests that the paused 30S PIC presents the mRNA for targeted recognition and degradation. Ribonuclease activity on PIC-bound *ompD* is critically dependent on the recruitment of RNase E into the multi-enzyme RNA degradosome, and our data suggest a process of substrate capture and handover to catalytic sites within the degradosome, in which sequential steps of seed matching and duplex remodelling contribute to cleavage efficiency. Our findings support a putative mechanism of surveillance at translation that potentially terminates gene expression efficiently and rapidly in response to signals provided by regulatory RNA.

## Graphical Abstract

**Graphical Abstract:**
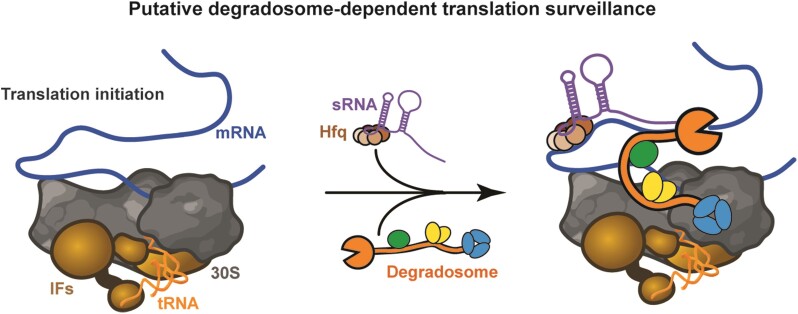

## Introduction

Small regulatory RNAs (sRNAs) contribute widely to the control of gene expression in phylogenetically diverse bacteria and enrich the repertoire, complexity, and stability of the post-transcriptional regulatory networks that govern much of their behaviour (1–4). Ranging from roughly 50 to 200 nucleotides in length, many sRNAs act *in trans* using short ‘seed’ regions that recognize target transcripts through oftentimes imperfect base-pairing. In most of these cases, the physical interaction between the sRNA and the cognate transcript results in translational repression, often with an ensuing degradation of the mRNA (5). In gamma-proteobacteria, the enzyme responsible for most sRNA-triggered degradation events is the conserved endoribonuclease E (RNase E) (6–8). Cleavage by RNase E appears to commit the sRNA-tagged transcript to a fate of rapid and complete degradation.

RNase E recognizes and cleaves substrates through two proposed pathways: (i) a route in which recognition of a monophosphate at the 5′-end of an RNA allosterically activates the enzyme and (ii) a route of internal entry, which requires recognition of the substrate fold (9–13). These pathways can potentially cooperate so that a duplex formed between two RNA molecules or within one molecule can satisfy the requirements of a single-stranded monophosphorylated 5′-end delivered for 5′ recognition, as well as having a single-stranded region that would fit along the shallow groove at the active site. Recent structural data indicate how duplex regions in an RNA substrate can be recognized by the catalytic domain of RNase E to facilitate presentation of single-stranded regions to the active site for cleavage (9,14).

Accumulating evidence indicates that the C-terminal portion of RNase E outside the catalytic domain also plays an important role in supporting degradation and sRNA-mediated regulation. This C-terminal portion serves as a scaffold for a multi-enzyme assembly known as the RNA degradosome and is predicted to be predominantly in an intrinsically disordered form but carries micro-domains mediating recognition of the protein partners, which in gamma-proteobacteria include a DEAD-box RNA helicase RhlB, the glycolytic enzyme enolase, and the exoribonuclease polynucleotide phosphorylase (PNPase) (15,16). Two RNA-binding domains flank the helicase binding site, and in addition the C-terminal domain carries a membrane-association motif that targets the assembly to the cytoplasmic membrane (17–20). The location of the degradosome on the membrane in gamma-proteobacteria places restrictions on access to substrates such as the nascent transcripts exiting the RNA polymerase that might reside in the physically distant nucleoid (18).

*In vivo*, truncation of RNase E to disrupt the degradosome assembly (21,22) or membrane association (23) impacts the kinetics or efficiency of substrate cleavage. Removal of the RNase E C-terminal domain diminishes the efficiency of co-degradation of the sRNA-mRNA pair RyhB-*sodB*, implying that the degradosome might be important for presenting the sRNA-tagged substrate to the catalytic domain of RNase E (24,25). Moreover, the truncation is synthetically lethal when combined with mutations in the 5′-end sensing pocket of the catalytic domain, suggesting that the pathways of degradation involving 5′-end activation and direct-entry are non-redundant and that there is an interplay between the C- and N-terminal domains during substrate recognition and cleavage (26).

To explore the process by which sRNAs mediate silencing through RNase E, we studied the sRNA MicC that directs cleavage and turnover of the *ompD* transcript. Assisted by the RNA chaperone Hfq, MicC recognizes *ompD* and acts as a *trans* activator to induce downstream *ompD*-cleavage by RNase E (27–29). Considering that *ompD*-targeting occurs in the coding region (codons 23–26) and that the ternary complex of MicC and Hfq on *ompD* does not prevent ribosome engagement or translocation (28), the question arises whether the regulation by the sRNA could occur at the stage of translation pre-initiation complex (PIC) formation on the 30S ribosomal subunit.

In this work, we observe that the 30S translation PIC can present the region of the *ompD* transcript that is complementary to the MicC seed to regulate downstream mRNA cleavage by RNase E, as well as boost the inactivating co-cleavage of the sRNA. When *ompD* is presented by the PIC, the sRNA-mediated deactivation of *ompD* cannot be carried out efficiently by the isolated catalytic domain of RNase E but requires the degradosome assembly. Partial melting of the sRNA-mRNA duplex is anticipated to be necessary to present the 5′-terminus of the sRNA to the 5′-sensing pocket of RNase E, and our data for variant seed pairing strengths support this possibility. The presented data supports a model in which the degradosome-scaffolding region of RNase E facilitates this action on the sRNA in the process of substrate capture and channeling to the nuclease's active site and further suggests that the 30S PIC regulates the ability of RNase E to gain access to the activating 5′ phosphate group on the sRNA.

## Materials and methods

### Protein preparation

*Escherichia coli* Hfq, RNase E catalytic domain (residues 1–529) and RNA degradosome were prepared using previously described procedures (9,30,31). The degradosome preparation includes RNase E, RhlB, enolase and polynucleotide phosphorylase (PNPase). Wild-type 30S subunits were prepared from *E. coli* MRE600 using zonal centrifugation (32,33). Initiator tRNA (fMet-tRNA^fMet^) was purified using HPLC and *E. coli* initiation factors IF1, IF2 and IF3 were purified following published protocols (34).

### RNA preparation

RNAs were synthesized by *in vitro* transcription (IVT) as described (27). In brief, IVT templates were amplified by PCR from plasmid pVP042 that carries the *Salmonella* Typhimurium *ompD* gene (28). The forward primer introduces a T7 promoter sequence upstream of the transcription initiation site. IVT reactions containing 3 μg of template DNA, 5 mM of each of ATP, UTP, GTP and CTP, 10 mM DTT (dithiothreitol) and 0.5 U/μl RNaseOUT™ (Invitrogen) were incubated with purified recombinant T7 RNA polymerase in 40 mM Tris, pH 8.0, 25 mM MgCl_2_, 2 mM spermidine at 37°C for 5 h. IVT products were DNase I-treated, resolved by 4% denaturing PAGE and excised RNA bands were electroeluted using an Elutrap™ Electroelution System Kit (Whatman), followed by separation using a PureLink™ RNA Micro Kit (Invitrogen).

### Isothermal titration calorimetry measurements

Isothermal titration calorimetry (ITC), used to quantify RNA annealing, was performed using a MicroCal ITC200 microcalorimeter (Malvern Panalytical). *ompD* and MicC 12mers (Dharmacon) were buffer exchanged in 25 mM Tris pH 7.5, 50 mM NaCl, 50 mM KCl, 10 mM MgCl_2_, 1 mM DTT using Micro Biospin 6 Chromatography Columns (Bio-Rad). ITC measurements were performed at 30°C with stirring at 1000 rpm. Following an initial injection of 0.4 μl, injections of 2 μl MicC 12mer variants into 12mer *ompD* were separated by 100 s to allow the system to equilibrate between injections. Data were analyzed using the ORIGIN software (OriginLab).

### Preparation of *ompD*-PIC

30S PIC was prepared following a previously described strategy (35). In brief, 30S subunits were incubated in buffer TAKM_20_ (50 mM Tris–HCl pH 7.5, 70 mM NH_4_Cl, 30 mM KCl, 20 mM MgCl_2_) for 30 min at 37°C for reactivation. Reactivated 30S subunits were incubated with a 2.5-fold excess of mRNA, 2-fold excess of initiation factors IF1, IF2 and IF3, and a 2.5-fold excess of initiator fMet-tRNA^fMet^ (hereafter tRNA) in the presence of 250 μM GTP or GTPγS (Jena Bioscience) in TAKM_7_ buffer (35). The complete complex was purified from the free components by size exclusion chromatography using a BioSEC5-1000 A matrix (Agilent), and the assembly was corroborated by denaturing protein and RNA electrophoresis gels. A fraction containing all components was used for cleavage assays, directly following purification.

### RNA degradation assays

Degradation assays were carried out in degradation buffer (25 mM Tris–HCl, pH 7.6, 50 mM NaCl, 50 mM KCl, 10 mM MgCl_2_, 2 mM DTT, 0.5 U/μl RNaseOUT™) as described previously (27). Substrates (free *ompD*, *ompD*-PIC) were prepared as described above and pre-incubated with indicated MicC sRNA or 12-mer variants and Hfq at 37°C, at final concentrations of 0.2 μM if not otherwise indicated. Reactions were started by addition of the enzyme at indicated final concentrations. Reaction aliquots were withdrawn at indicated time points and quenched with equal volume stop buffer at 50°C for 30–45 min. Samples were resolved on 8–10% denaturing polyacrylamide gels, stained in SYBR Gold (Invitrogen) and visualized under UV light. Bands were quantified with GeneTools or ImageJ and plotted with GraphPad PRISM. Degradation rates of full-length sRNAs and mRNA segments were determined by fitting the data to the one phase decay fit equation in GraphPad PRISM, [*I* = (*I*_0_ – *P*) × e^-^*^kt^*) + *P*] with relative abundance, *I*; plateau, *P*; degradation rate, *k*; reaction time, *t*. Turnover rates of intermediates were determined by fitting the relative abundance to two-exponential equation [*I* = e^-^*^kt^* – e^-^*^jt^*] with relative abundance, *I*; degradation rate, *k*; formation rate, *j*; reaction time, *t*. Cleavage site specificities were calculated by dividing initial intermediate formation rates by the respective RNA degradation rate. Preference multiples were determined by dividing specificities for sites +83 and +72 of individual reactions. Reactions with free *ompD* were carried out in at least triplicates, reactions with *ompD*-PIC were done with triplicates for the degradosome (all MicC variants) and RNase E (1–529) (MicC WT, W1 and S1).

### Construction of plasmids and bacterial strains

Plasmids expressing MicC variants were constructed by cleavage of the plasmid pKP21-13 encoding wild type MicC with BsaXI (Weaker 1, 2 and 3) or BamHI/HpaI (Stronger 1, 2 and 3), and replacement of the removed fragment with hybridized oligonucleotides introducing the desired mutation: W1for/W1rev (pBAD-MicC-W1), W2for/W2rev (pBAD-MicC-W2), W3for/W3rev (pBAD-MicCW3), S1for/S1rev/MicCStrongerFor/MicCStrongerRev (pBAD-MicC-S1), S2for/S2rev/MicCStrongerFor/MicCStrongerRev (pBAD-MicC-S2) and S3for/S3rev/MicCStrongerFor/MicCStrongerRev (pBAD-MicC-S3) (Table 1).

All strains unsed in this study are listed in Table 2. Phage P22 transduction was employed to transfer each single chromosomal modification to a fresh *Salmonella* wild-type background and to obtain strains carrying multiple mutations. To eliminate the Kan^R^ cassette of λRed-derived mutants, cells were transformed with the FLP recombinase expression plasmid pCP20 (36). Mutant susceptibility to kanamycin and loss of the temperature-sensitive FLP expression plasmid were tested.

**Table 1.** Oligonucleotides

Name	Sequence 5′-3′	Application
JVO-0322	CTACGGCGTTTCACTTCTGAGTTC	hybridization 5S rRNA
JVO-0718	GAAAGCAAAGGTTAACGCAATG	hybridization MicC sRNA
JVO-4314	TGCCACTAACTTAAGTTTCAT	hybridization *ompD* mRNA
W1for	CAACTCTCTACTGTTTCTCCATTATATGCC	plasmid construction
W1rev	ATATAATGGAGAAACAGTAGAGAGTTGCGA	plasmid construction
W2for	CAACTCTCTACTGTTTCTCCGCTATATGCC	plasmid construction
W2rev	ATATAGCGGAGAAACAGTAGAGAGTTGCGA	plasmid construction
W3for	CAACTCTCTACTGTTTCTCCGTCATATGCC	plasmid construction
W3rev	ATATGACGGAGAAACAGTAGAGAGTTGCGA	plasmid construction
MicCStrongerFor	GATCCTACCTGACGCTTTTTATCGCAACTCTCTACTGTTTCTCC	plasmid construction
MicCStrongerRev	AACGCAATGGCCCAGCGACAAAATGAATATGT	plasmid construction
S1for	GTTATATACCTTTATTGTCACATATTCATTTTGTCGCTGGGCCATTGCGTT	plasmid construction
S1rev	GACAATAAAGGTATATAACGGAGAAACAGTAGAGAGTTGCGATAAAAAGCGTCAGGTAG	plasmid construction
S2for	GTTATATGCCTCTATTGTCACATATTCATTTTGTCGCTGGGCCATTGCGTT	plasmid construction
S2rev	GACAATAGAGGCATATAACGGAGAAACAGTAGAGAGTTGCGATAAAAAGCGTCAGGTAG	plasmid construction
S3for	GTTATATACCTCTATTGTCACATATTCATTTTGTCGCTGGGCCATTGCGTT	plasmid construction
S3rev	GACAATAGAGGTATATAACGGAGAAACAGTAGAGAGTTGCGATAAAAAGCGTCAGGTAG	plasmid construction

### RNA isolation and northern blot analysis

*Salmonella* strains Δ*micC*, Δ*micC rne701* and Δ*micC* Δ*rnc* carrying either a control plasmid (pBAD*-ctrl*.) or MicC expression vectors (pBAD-MicC, pBAD-MicC-S1, pBAD-MicC-S2, pBAD-MicC-S3, pBAD-MicC-W1, pBAD-MicC-W2, pBAD-MicC-W3) were grown aerobically at 37°C in LB broth supplemented with ampicillin (100 μg/ml) to mid-exponential phase (OD_600_ of 0.6) when expression from the pBAD promoter was induced by addition of arabinose (0.1% final concentration). Total bacteria samples were collected, mixed with 0.2 volumes of stop-mix (95% ethanol and 5% phenol, v/v) and snap-frozen in liquid nitrogen. Total RNA was isolated using the Hot Phenol method as described previously (37). For Northern blot analysis, 5 μg of total RNA were separated on 5% polyacrylamide (7 M urea) gels in 1xTBE buffer and electroblotted. Membranes were hybridized with gene-specific P^32^ 5′ end-labelled DNA-oligonucleotides (MicC: JVO-0718; *ompD* mRNA: JVO-4314; 5S rRNA: JVO-0322) at 42°C in Roti-Hybri-Quick hybridization solution (Roth) and washed in three subsequent steps with SSC wash buffers (5×/1×/0.5 × SSC) supplemented with 0.1% SDS and imaged with a Typhoon FLA 7000 (GE Healthcare) phosphor-imager.

**Table 2.** *Salmonella* Typhimurium strains

Trivial name	Stock	Genotype	Reference
SL1344	JVS-00007	*StrR hisG rpsL xyl*	laboratory stock
	JVS-03387	SL1344 Δ*micC*	Pfeiffer *et al.* (2009)
	JVS-01238	SL1344 *rne701::Kan^R^*	Pfeiffer *et al.* (2009)
	JVS-00938	SL1344 Δ*rnc::Kan^R^*	Viegas *et al.* (2007)
	JVS-11859	SL1344 Δ*rnc*	This study
Δ*micC*	JVS-00051	SL1344 Δ*micC::Kan^R^*	Papenfort *et al.* (2008)
Δ*micC rne701*	JVS-11358	SL1344 Δ*micC rne701::Kan^R^*	This study
Δ*micC* Δ*rnc*	JVS-12440	SL1344 Δ*micC::Kan^R^* Δ*rnc*	This study

## Results

### The RNA degradosome can access mRNA in the translation pre-initiation complex

Earlier studies of RNase E activity on the *ompD* transcript *in vivo* identified position +83 in the coding region as a preferred cleavage site when the sRNA MicC is expressed (27,28). *In vitro*, RNase E cleaves *ompD* at additional sites downstream of the +83 site, probably due to changed accessibility of this fragment in the *in vitro* system; however, the +83 cleavage is present only in the presence of MicC and is substantially boosted when the sRNA carries a 5′ monophosphate (27). Moreover, in the absence of MicC a cleavage site at position +72 is observed, which is within the MicC*-ompD* pairing region, and is protected from RNase E activity when the sRNA-mRNA pairing occurs. For the following experiments, we are concentrating on the efficiency of the +83 cleavage as the reflection of the *in vivo* degradation pattern, and on the absence of cleavage at position +72, which is a marker of efficient sRNA binding.

**Figure 1.**
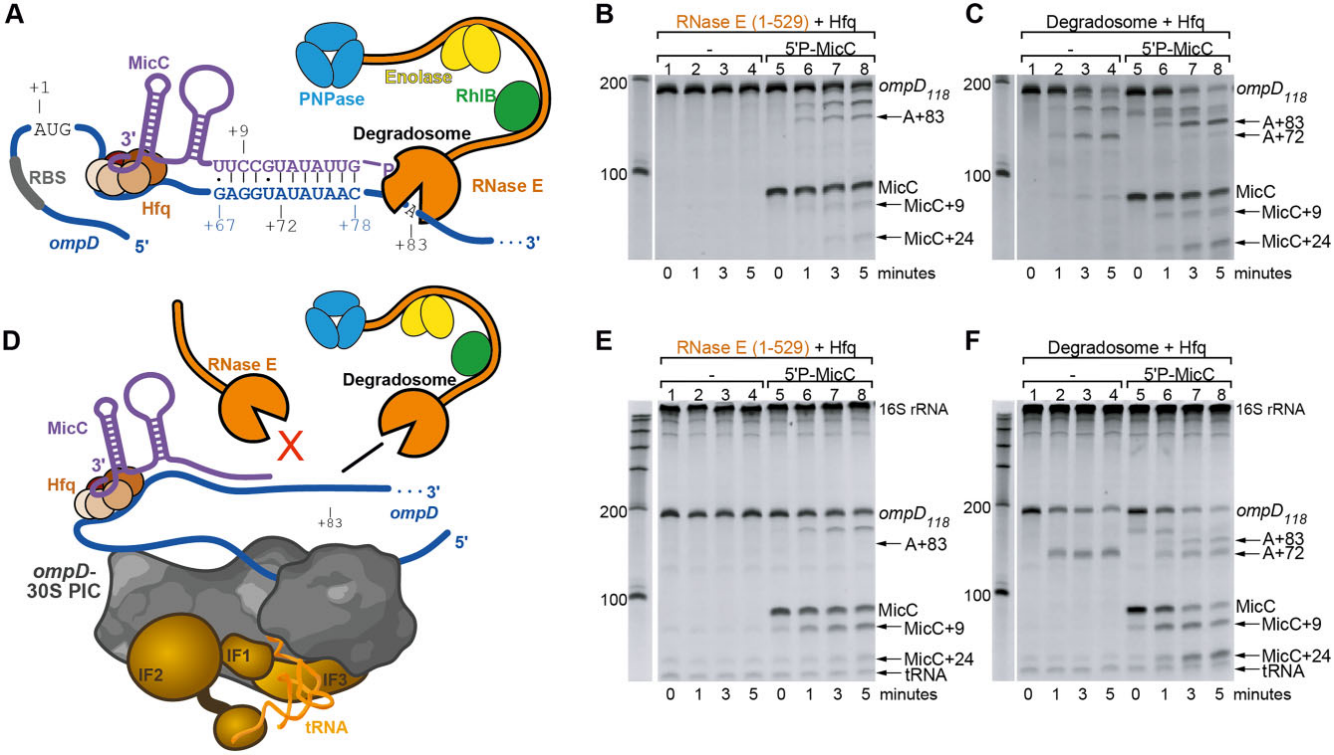
MicC-guided cleavage of the *ompD* mRNA by RNase E dependent on the RNA degradosome assembly when *ompD* is engaged in the 30S PIC. (**A**) Schematic model for the ternary complex of MicC, Hfq and the *ompD* transcript guiding *ompD*-cleavage by the RNA degradosome to position +83. Additional cleavage sites +72 on *ompD* and +9 on MicC are marked. (**B, C**) *In vitro* cleavage patterns of free 200 nM *ompD_118_* by 200 nM RNase E (1–529) (B) or by 44.2 nM full RNA degradosome (C). (**D**) Schematic of the 30S PIC bound to the ribosome binding site (RBS) of *ompD* presenting the downstream cleavage sites within the ternary complex. (E, F) *In vitro* cleavage patterns of *ompD_118_*-PIC by 200 nM RNase E (1–529) (**E**) or by the 44.2 nM full RNA degradosome (**F**). Digests were performed in the presence or absence of the 200 nM full length 5′-monophosphorylated MicC sRNA and Hfq, as indicated above the gel images. The +83- and +72-products from *ompD* cleavage as well as +9 and +24-cleavage products of MicC cleavage are indicated with arrows. Time-point 0 was taken immediately after enzyme addition (about 10 s). The buffer used for assays does not support PNPase activity (30,38), therefore all degradation events are the result of RNase E activity.

The cleavage site in *ompD* is downstream of the base-pairing region between the mRNA and the sRNA, which suggests that the RNA-RNA interaction helps to align RNase E to attack the substrate (Figure 1A). Consistently, when an *ompD* mRNA fragment that encompasses the 69 nts long 5′ UTR and the first 118 nts of the coding region (*ompD_118_*) is incubated with the highly purified RNase E catalytic domain (RNase E 1–529) *in vitro*, 5′P-MicC induces cleavage at the +83 site (9,27) (Figure 1B; Supplementary Figure S1). The level of intact MicC decreases over the course of the reaction, giving rise to deactivated MicC species that are cleaved at positions +9 and/or +24 and therefore lack the seed region (27) (Figure 1B). The full RNA degradosome assembly also produces this expected cleavage pattern with the +83-cleavage intermediate in the presence of 5′P-MicC (Figure 1C; Supplementary Figure S1). The +72-cleavage intermediate corresponds to degradosome cleavage within the region of complementarity to the MicC seed sequence and, in the absence of MicC, is the dominant intermediate species (Figure 1C, lanes 2–4). The *in vitro* reaction conditions do not support cleavage by the exoribonuclease PNPase as they do not contain inorganic phosphate (30,38); therefore, all degradation events are expected to be the result of RNase E activity.

To explore if the *ompD* transcript remains accessible to RNase E activity in the early stages of translation, we examined how the 30S PIC might impact cleavage of the mRNA. The *ompD_118_* fragment was assembled with purified 30S ribosomal subunit, initiator fMet-tRNA^fMet^, initiation factors IF1, IF2, IF3 and GTP to form the *ompD*-PIC (Figure 1D) which was subsequently separated from the free components by size exclusion chromatography using a BioSEC-5 1000 Å matrix and used for cleavage assays directly following purification.

When associated with PIC components, the *ompD* mRNA was inefficiently processed by the isolated catalytic domain of RNase E, which is unable to access the +83-cleavage site in the presence of MicC (Figure 1E; Supplementary Figure S1). In contrast, the full RNA degradosome assembly can process *ompD* when the mRNA is engaged with the 30S PIC (Figure 1F; Supplementary Figure S1), producing +83-cleavage intermediates in the presence of MicC. This result indicates that the interaction of the C-terminal domain of RNase E and/or the other degradosome components with the 30S subunit modulates the nuclease's access to the sRNA-induced cleavage site in the transcript and therefore could play a role in gene expression regulation. Notably, in the presence of the 30S PIC, intact MicC levels decrease in parallel with that of the *ompD* transcript, whereas in the absence of the PIC, the sRNA is degraded more slowly than *ompD*. This suggests that the PIC might expose cleavage sites on both the sRNA and mRNA and may facilitate synchronized degradation of the two RNAs by the RNA degradosome. As stable complexes can form between the 30S subunit and the RNA recognition core of the degradosome, comprising the non-catalytic segment 603–850 from RNase E, RhlB helicase and enolase (31,39,40), PIC assembled on *ompD* might be also interacting with the C-terminal domain of RNase E and/or other degradosome components directly. This interaction could play a role in regulating access to cleavage sites on an mRNA that is engaged in the 30S PIC.

### RNase E cleavage efficiency and specificity are influenced by the strength of the sRNA–mRNA duplex

**Figure 2.**
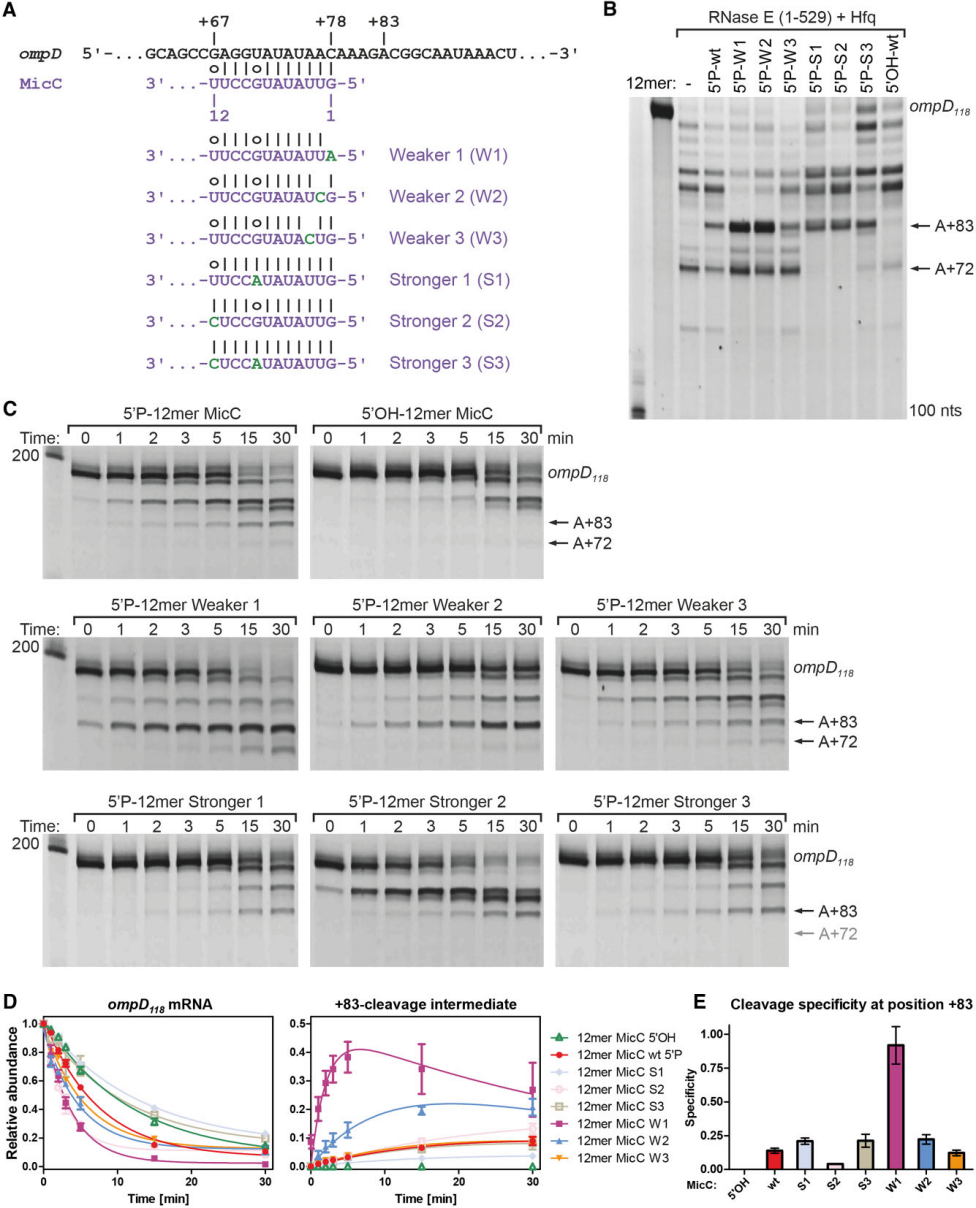
The influence of the strength of the sRNA-mRNA duplex on cleavage by RNase E catalytic domain (RNase E (1–529)). (**A**) The fragment of *ompD* sequence (black) encompassing the MicC seed-binding site and the sRNA-mRNA pairing (purple; top panel). The sequences of the seeds (purple) with the mutations (green) influencing the strength of the MicC-*ompD* interaction are indicated. (**B**) RNase E degradation of 400 nM *ompD_118_* with 200 nM RNase E (1–529) in the presence of 600 nM MicC 12mers. Reactions were incubated for 30 minutes. **(C)** Time course reactions of 200 nM *ompD_118_* degradation with 150 nM RNase E (1–529) in the presence of different MicC seed variants (each at 300 nM). The reactions were stopped at the indicated time points (0, 1, 2, 3, 5, 15, 30 min). Time point 0 was taken immediately after enzyme addition (about 10 s). Grey arrow labeled A +72 points to the expected +72 cleavage product absent for stronger MicC mutants. (**D**) Relative abundances of the full-length *ompD_118_* fragment and the +83-cleavage intermediate over time from experiments in C. (**E**) Specificities for cleavage at the +83 site in the presence of the respective MicC 12mer variants, determined as above. Data are mean ± SD from three independent reactions.

It is anticipated that an sRNA can be an effective *trans*-activator of RNase E if the molecule can provide an accessible monophosphate group that can bind to the 5′-end sensing pocket of the enzyme and boost the catalytic activity. Many *trans*-encoded sRNAs bear a seed region at the very 5′-end (41–43) and can therefore engage in duplex formation with a target such that it might be available to activate RNase E allosterically. Accordingly, if the duplex formed by sRNA and mRNA is recognized by RNase E, it must melt at least partially for the 5′-end of the sRNA to be accommodated in the enzyme's 5′-end sensing pocket. To test this hypothesis, and to explore the impact on activity within the PIC, the seed region of MicC was altered to change its interaction free energy with *ompD*. Wild type MicC forms an imperfect 12 bp duplex with the target sequence in *ompD* (14). Six different MicC seeds were designed with different predicted energies for binding *ompD*: three ‘weaker’ and three ‘stronger’ compared to wild type MicC (Figure 2A). To confirm the ranking for duplex stability, the free energies for pairing between MicC variants and *ompD* were quantified experimentally using isothermal titration calorimetry (Supplementary Table S1).

The MicC series was first tested as 12mers encompassing the seed region for their capacity to guide RNase E catalytic domain (RNase E (1–529)) to cleave *ompD*. All 12mers harbored a 5′-monophosphate group to activate RNase E cleavage *in trans*, and while each can induce +83-cleavage of *ompD* by RNase E (1–529), the cleavage efficiency differs for the various MicC seeds (Figure 2B). Remarkably, when the duplex between sRNA and mRNA was perturbed by substitutions in the first or second position from the 5′-end of the seed region, the cleavage at position +83 becomes more efficient compared to the wild type MicC seed (Figure 2B, Weaker 1 and 2). When the mutation in the seed region was in the third position from the 5′-end, the sRNA-12mer behaved closer to wild type (Figure 2B, Weaker 3), which suggests that the first two nucleotides of the seed are the most important in influencing the speed and efficiency of RNase E action *in vitro*. This agrees with the crystal structure of RNase E in complex with a fragment of MicC sRNA (9), in which only the first two terminal nucleotides of the RNA are engaged with the enzyme: the first nucleotide is buried in the 5′ binding pocket, whereas the second one interacts with R141. To form these interactions, the 5′-terminal nucleotides must be free and readily peeled from the mRNA.

The influence of the MicC seed variants in mediating RNase E (1–529) cleavage was investigated further in time course reactions to quantify the effect of the different MicC seeds on *ompD* degradation (Figure 2C, D). The weaker MicC 12mer mutants caused between 1.3- and 2-fold faster *ompD* degradation. Formation rates of +83-cleavage intermediates showed marked increase in the presence of Weaker 1 (13-fold) and Weaker 2 (2.5-fold), supporting the above-described observations. Comparing formation rates for +83 intermediates in proportion to respective *ompD* degradation rates shows that especially the Weaker 1 seed drastically increased RNase E preference for the +83 site (Figure 2E). The efficiency of the +83-cleavage in the presence of the stronger seeds is comparable to that of the wild type MicC 12mer; however, the overall *ompD* degradation is slower when the stronger substitution is introduced in the 8th position from the beginning of the seed region or when combined substitutions are made at both the 8th and 12th positions (Figure 2C, D, Stronger 1 and 3). These substitutions seem to trap the enzyme in a non-active conformation, which is consistent with the hypothesis that the sRNA-mRNA duplex must melt to facilitate RNase E action. Surprisingly, the fastest degradation rate for *ompD* was observed in the presence of the Stronger 2 seed, where a cleavage product larger than the +83-intermediate accumulated (Figure 2C). The differences in *ompD* degradation rates suggests that fold recognition might be another aspect of RNase E action on the sRNA-mRNA duplex, in addition to duplex strength.

Consistent with the observations made for full-length MicC, the +72 site of *ompD* that is accessible to RNase E attack in the absence of the sRNA also becomes protected in the presence of the various MicC 12mer variants. The abundance of the cleavage product from the +72 site increases as the sRNA-mRNA interaction becomes weaker, while the stronger MicC seed mutants confer greatest protection (Figure 2C).

**Figure 3.**
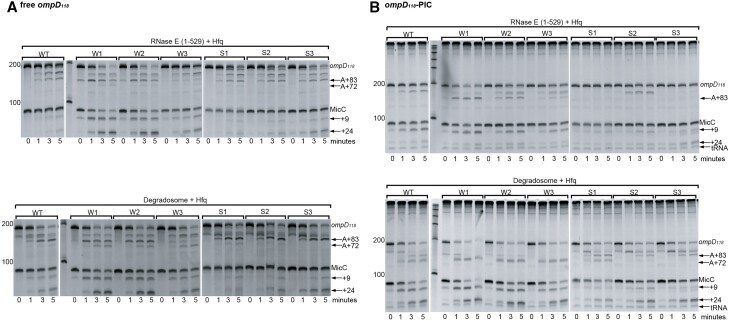
The influence of the strength of the sRNA-mRNA duplex on cleavage by RNase E and the degradosome in free *ompD* and the *ompD*-PIC complex. (**A**) 200 nM *ompD_118_* degradation by 200 nM RNase E (1–529) (top panel) or the 44.2 nM RNA degradosome (bottom panel) in the presence of 200 nM Hfq and MicC with different indicated seed variants: W1, W2, W3, S1, S2 and S3 correspond, as shown in Figure 2A. (**B**) *ompD_118_*-PIC degradation by 200 nM RNase E (1–529) (top panel) or the 44.2 nM RNA degradosome (bottom panel) in the presence of 200 nM Hfq and MicC with different indicated seed variants. Time point 0 was taken immediately after enzyme addition (about 10 s).

The observation that seeds with readily peeled 5′ nucleotides boost the efficiency of *ompD* deactivation supports a model in which the two 5′-terminal nucleotides of the MicC seed must be unwound to activate RNase E in *trans*. To assess possible cooperative involvement of components within the RNA degradosome, we compared the activity of recombinant degradosome and RNase E (1–529) towards *ompD* in the presence of full-length monophosphorylated MicC with the different seed variants (Figure 3A; Supplementary Figure S1). The degradosome reactions are very similar to those with RNase E (1–529), with Weaker 1 and 2 boosting RNase E activity leading to accelerated +83 cleavage and increased MicC co-deactivation, whereas stronger seeds impede MicC co-degradation (Figure 3A; Supplementary Figure S1).

The series of weaker/stronger MicC constructs was also tested for the cleavage activity of RNase E catalytic domain (1–529) and the degradosome on the purified *ompD_118_*-PIC complex (Figure 3B; Supplementary Figure S1). As described above, isolated RNase E catalytic domain, in contrast to the full degradosome, was unable to cleave the *ompD*-PIC at position +83 in the presence of wild type MicC (Figure 1E). While this is also the case in the presence of stronger MicC variants (Figure 3B, upper panel, S1, S2, S3), RNase E catalytic domain can cleave the *ompD*-PIC at position +83 when the duplex of sRNA and mRNA is weakened at the 5′-end of the sRNA seed (Figure 3B, upper panel, W1, W2, W3). In contrast, the RNA degradosome cleaves the *ompD*-PIC at the position +83, also in the presence of the stronger MicC seeds, which indicates that the degradosome assembly supports RNase E activity on PIC-engaged mRNA. Moreover, as the isolated catalytic domain can access the MicC-*ompD* duplex when not engaged on PIC, but is unable to cleave *ompD* in the presence of WT and stronger MicC variants within PIC, 30S-PIC assembly might restrict access of RNase E to the sRNA–mRNA duplex, but this restriction can be overcome in the presence of the other degradosome components.

In experiments with the full degradosome, the presence of the weaker MicC variants in reactions with *ompD*-PIC resulted mostly in +72-cleavage intermediates, whereas these same seeds provided more protection from +72-cleavage in the absence of the PIC (Figure 3A, B, compare lower panels). The +72 cleavage might be also a result of further degradation of the +83 intermediate, and therefore might be arising from faster progression of the reaction. With wild type or stronger pairing, separation of the MicC seed and complementary *ompD* fragment requires more energy than when weaker MicC variants are used. This separation might be facilitated by the PIC, but only when the whole degradosome is present. Moreover, all MicC variants were cleaved faster when *ompD* was engaged in the PIC (compare MicC bands between Figure 3A and B lower panels), and the trend is more pronounced for the weaker series, indicating that the sRNA pairing with the mRNA must be disrupted for RNase E cleavage to continue. These findings support a model in which the PIC assists the degradosome in remodeling the sRNA–mRNA duplex during or following +83-cleavage causing faster and/or complete sRNA-mRNA duplex melting, which results in the access to the +72-cleavage site on *ompD* and presenting MicC cleavage sites for sRNA-deactivation.

### MicC-*ompD* duplex pairing strength affects *ompD* degradation *in vivo* and requires the non-nucleolytic components of the RNA degradosome

The impact of varying MicC seed strength on *ompD* decay was also investigated *in vivo*.

**Figure 4.**
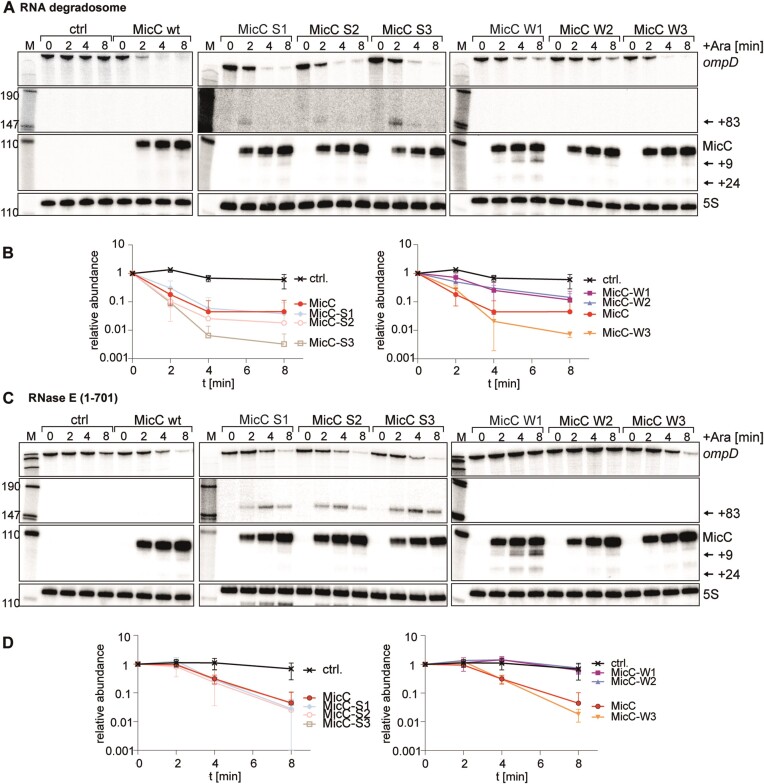
The influence of the degradosome assembly and MicC seed strength on *ompD* degradation *in vivo*. Northern blot for MicC wt, weaker and stronger series in extracts from *Salmonella* cells where expression was induced from an P_BAD_ promoter or a control plasmid. Samples were obtained before and 2, 4 and 8 min after addition of L-arabinose. Processed RNA species are indicated by arrows. Probing for 5S serves as loading control. Representative northern blots for *ompD* upon pulse expression of indicated MicC sRNA variants in strain, and plots of signal for *ompD* full-length species from the triplicates of the experiment with the wild type degradosome assembly (**A**, **B**) or RNase E (1–701) (**C**, **D**). Cleavage of *ompD* transiently accumulates the +83 intermediate.

MicC wild type and weaker/stronger variants were individually expressed from a pBAD vector in strains of *Salmonella* Typhimurium lacking chromosomal *micC* (Figure 4A). Following induction, cell extracts were prepared in a time series and MicC and mRNA fragments of *ompD* were detected by northern blot analysis (Figure 4). Upon expression of the wild-type sRNA (Figure 4B), a bi-phasic response was observed, with an initial rapid decrease in *ompD* transcript levels to less than 10% of the starting levels within 4 min, followed by a slower decay (Figure 4B, D). All MicC variants were expressed within the first minutes of induction and capable of mediating *ompD* degradation *in vivo*. Unexpectedly, Weaker 1 and 2 were the least effective in triggering *ompD* degradation, while Stronger 2 and Stronger 3 were the most effective MicC variants. These observations stand in striking contrast to their behavior *in vitro* and suggest that additional processes and factors might be involved in defining the *in vivo* degradation rate that are not recapitulated with the purified *in vitro* conditions. For instance, the RNA-RNA interactions mediated by the weaker series might be disfavored by processes that prevent off-targeting due to mismatching, and that only a subset of sRNA-mRNA duplexes would induce degradation. For the stronger series, the sRNAs might trigger an alternative degradation pathway mediated for example by RNase III, which cleaves double-stranded RNA duplexes (44–46). In fact, MicC/*ompD* complexes with stronger base-pairing are better substrates for RNase III in *in vitro* assays (Supplementary Figure S2). In an RNase III-deficient strain of *Salmonella*, the Stronger 3 MicC variant has a similar impact on *ompD* as wild type MicC and accumulates to higher levels (Supplementary Figure S2).

To test if the degradosome organization contributes to the process of MicC-mediated degradation of *ompD*, the assays were repeated with cells expressing RNase E (1–701), which is a truncated version of RNase E that lacks the portion of the C-terminal domain encompassing the second RNA binding motif (AR2) and the interaction sites for enolase, helicase and PNPase, but retains the membrane-association signal and the first RNA binding microdomain. All MicC constructs, including the wild-type, exhibited a pronounced lag phase before the onset of *ompD* degradation (Figure 4D), suggesting that the C-terminal domain of RNase E supports enzyme activity in sRNA-mediated mRNA degradation, most likely through substrate capture. This is consistent with results of single molecule studies of the action of the sRNA SgrS on the *ptsG* transcript *in vivo*, where a truncation of RNase E that disrupts of the degradosome assembly impacts on the kinetics of substrate cleavage (21).

We noted that the +83-cleavage product accumulated transiently with the stronger seeds *in vivo*, while this intermediate was not detected for the wild type seed (Figure 4C). This suggests that the subsequent degradation steps *in vivo* are inefficient, which might arise because of product inhibition of RNase E, in which the enzyme is not able to separate the strong seed from its target to proceed with degradation. Given that the rate of decay of MicC on the PIC is dependent on the strength of the seed pairing (*in vitro*), the *in vivo* results may also reflect a process where sRNA lifetime is modulated upon meeting a target.

## Discussion

The results presented here provide insight into how RNase E and the RNA degradosome interact with a sRNA-tagged transcript for activated cleavage and suggest models for the physical process. Data from duplexes with different pairing strength suggest that the first two nucleotides of the sRNA seed are the most important in influencing the speed and efficiency of RNase E action *in vitro* (Figures 2 and 3). This is in accord with the crystal structure of RNase E in complex with a fragment of MicC sRNA (9), in which the first two terminal nucleotides of the RNA are engaged with the enzyme, where the first nucleotide is buried in the 5′ binding pocket and the second interacts with R141. To form these interactions, the 5′-terminal nucleotides must be free and readily peeled from the mRNA. The optimization of the duplex length and strength in bacterial sRNAs to improve the cellular functionality of these molecules resembles the recently described characteristics of the eukaryotic microRNAs, in which the efficiency of targeting is precisely regulated by base pairing with the target via the seed region, as well as flanking nucleotides (47). Moreover, it has been recently shown that mRNA degradation in eukaryotes is actively regulated also during translation, and this process is often driven by microRNAs (48). A similar regulation scheme could exist in bacteria.

**Figure 5.**
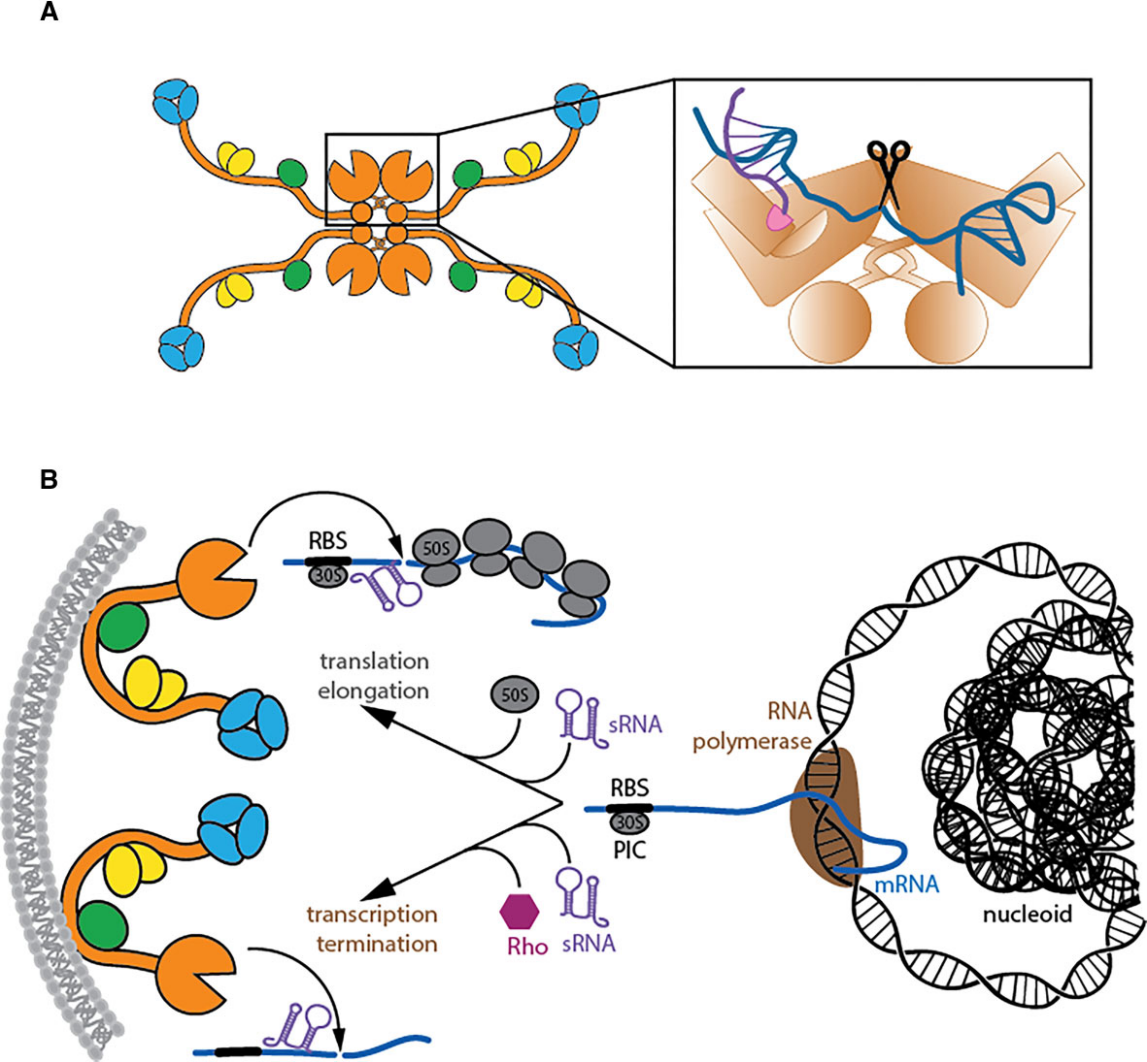
Working model for recognition of sRNA/mRNA pair by RNase E, and for the action of sRNA and the lifetime of the *ompD* transcript and possibly other mRNAs. (**A**) A combined mode of recognition of structured RNA by the S1/5′-sensor domain of RNase E (14) and the cryptic RNase H and KH folds (9). This mode might help in capturing substrates that are tagged by sRNA. (**B**) Substrate encounter in the cell. RNase E and the RNA degradosome are membrane-associated, but the nascent transcripts are in the nucleoid and may be physically distant. The degradosome and PIC could be brought into proximity during transertion (not shown). One mode of recognition of substrates envisages the mRNA-PIC on the leading edge of a polysome coming into proximity of the membrane, and activating RNase E by sRNA tagging. Another mode, which could operate in parallel, envisages sRNA-tagged transcripts being released from the RNA polymerase by the action of Rho helicase, and then diffusing until an encounter with the degradosome on the membrane.

We also envisage that the sRNA:mRNA duplex itself might act in early stages of substrate engagement. A mode of recognition of duplex RNA by the S1/5′-sensor domain of RNase E has been suggested based on recent cryoEM studies (14) (Figure 5A). Additionally, RNase E has another duplex recognition mode, involving the cooperation of the cryptic RNase H domain and the cryptic KH-domain fold that form the homotypic dimer interface of RNase E (9) (Figure 5A). We envisage that either or both modes can help in initial stages of substrate capture and handover to the catalytic site. Additionally, inter-domain cooperation is likely to involve handover of mRNA/sRNA that could be engaged by the RNA-binding segments within the C-terminal domain of RNase E and by other degradosome components or the RNA binding domains within the C-terminus of RNase E, particularly AR2 which has been implicated in sRNA action *in vivo* (49). The involvement of the degradosome in the efficient MicC-dependent *ompD* degradation is supported by *in vivo* results presented here, where truncated RNase E does not efficiently cleave the mRNA upon sRNA expression (Figure 4), as well as *in vitro* assays with the degradosome or the catalytic domain of RNase E, where the isolated catalytic domain cannot readily access the mRNA within the 30S translation pre-initiation (PIC) complex (Figure 1). Moreover, the described preference of RNase E for the sRNA–mRNA substrate is not the only determinant of the regulation outcome. The *in vivo* data indicate that weak interaction between the sRNA and the target, despite being favoured by the enzyme, does not increase the regulation. This could be a surveillance mechanism that protects the cell from off-target regulation by sRNAs that weakly bind to mRNAs that are not their true target, including competition with other sRNAs, and indeed the *ompD t*ranscript is known to be the target of many sRNAs *in vivo* (29,50). Conversely, if the pairing is strong, the sRNA:mRNA complex might become a target for an alternative degradation pathway initiated by RNase III, as occurs in *Staphylococcus aureus* (44,51,52). The interactions of sRNA with their targets could be optimized in the interaction position (presence of e.g. secondary structures that could be recognized by RNase E duplex recognition regions) and strength to achieve precision and speed required.

The experimental results presented here also indicate that the sRNA MicC can guide RNase E to cleave at a downstream site in the coding region of the *ompD* transcript at an early step of translation when that mRNA is engaged in the 30S PIC *in vitro*. The transcript presentation enables RNase E in the RNA degradosome to precisely cleave the mRNA at the sRNA-guided site (+83) in the coding region, after which the cleavage product is likely to be rapidly degraded further by the degradosome assembly. The co-degradation of the sRNA and mRNA is accelerated when the 30S PIC is present on the transcript. *In vivo* and *in vitro* data indicate that the degradosome-scaffolding region of RNase E facilitates the action of the sRNA, probably through substrate capture and channeling to the nuclease's active site, where partial melting of the sRNA-mRNA duplex helps to present the 5′-terminus of the sRNA to bind in the 5′-sensing pocket. Moreover, assembly of the degradosome on the C-terminal tail of RNase E is crucial for the interactions with other proteins and complexes involved in sRNA mediated gene regulation. The distal face of Hfq is known to prefer ARN motif arrays, and the *ompD* transcript harbours a 6xARN/RRN motif in coding region +15 to +32 which could serve as the binding site (53). Potentially, the sRNA presentation could be facilitated by interactions of the sRNA/Hfq with PNPase (30) and the RNA-binding segment of the RNase E C-terminal domain (39,49). After duplex formation, a chaperoning action by the PIC might aid the partial melting of the 5′-terminal seed base pairs to free the MicC 5′ monophosphate for RNase E activation. We envisage that this could occur at the PIC on the leading end of the transcript as it emerges from polyribosome assembly (Figure 5B). More experiments are necessary to precisely determine the sequence of events that happen during MicC mediated *ompD* silencing *in vivo*.

When the 30S ribosomal subunit forms the PIC on *ompD*, the processing of *ompD* at the MicC-guided cleavage site cannot be achieved efficiently by the isolated RNase E catalytic domain in *in vitro* degradation reactions but is dependent of RNase E as part of the RNA degradosome assembly. The previously observed interaction of the 30S ribosomal subunit and the recognition core within the C-terminal part of the degradosome underlines the potential interplay between translation and surveillance machines (31,39,40). Interestingly, the isolated RNase E catalytic domain was able to process *ompD* in the presence of MicC seed variants with weakened base pairing near the monophosphorylated 5′ end, which implies that the degradosome components assist in 5′ end melting of the seed and that the process is regulated by the presence of the PIC. Notably, structural investigations of the *ompD*-PIC (Roske et al., in preparation) identify density that is likely caused by the OB domains of the ribosomal S1 protein where it binds the A/U-rich segment that precedes the Shine-Dalgarno sequence as previously suggested (54). S1 assists the remodelling of mRNA secondary structures *in vitro* to aid the 30S with mRNA loading and PIC formation (55,56) and has also shown direct interaction with several sRNAs from *E. coli* (57), thereby possibly exerting the PIC’s influence on RNase E attack while located on the 30S ribosome after *ompD*-PIC assembly. S1 has been shown to bind RNAP and could aid in a putative RNAP-Hfq interaction (58,59), and this could facilitate the interplay with the PIC. More data are needed to explore this hypothetical interplay.

Our earlier data indicate that the RNase E-activating effect is achieved by the 5′ end of MicC, which stimulates the enzyme cleavage of *ompD* downstream of the sRNA-mRNA duplex (27). As the 5′P-MicC is highly unstable and only a small fraction of the endogenous sRNA was found to harbour 5′ monophosphate, it was suggested that the sRNA is activated by the 5′ diphosphate removal directly before RNase E recruitment, possibly after pairing with *ompD* (27). Recent data however suggested that the 5′ monophosphate is not a significant player in sRNA mediated regulation of gene expression, where overexpressed MicC was shown to cause *ompD* mRNA degradation regardless of the status of its 5′ end (60). The presented data do not exclude the possibility that the 5′-end activation mechanism proposed in (27) occurs within the cell. The analysis of the levels of overexpressed MicC in 5′PPP and 5′P forms in (60) suggests that the 5′PPP MicC is very stable, however a fraction could be converted to the 5′P form as the Northern blots revealed a MicC degradation product arising from the +9 cleavage by RNase E, which is present *in vitro* only for the monophosphorylated sRNA, and absent when 5′PPP-MicC was used (27). This raises the possibility that, despite overexpression of 5′PPP MicC, the *ompD* cleavage is activated by the processed, 5′P-MicC. Overexpression of 5′P-MicC on the other hand causes its rapid degradation, therefore only a small fraction of the sRNA can reach its target. Both studies tested the MicC mediated regulation in the Δ*rppH* strain, where the best studied Nudix protein in *E. coli* has been removed, and it was shown that one or more of the 13 Nudix proteins from this bacterium could have a redundant function important for MicC action. Therefore, the determination of the precise mechanism of this regulation still awaits determination.

An interesting issue arises around sRNA recruitment when considering the cellular location of the degradosome. As the RNA degradosome is bound to the inner membrane in gamma-proteobacteria, it could be physically separated from its substrates that are being transcribed in the nucleoid. It is possible that the mRNA–PIC complex on the leading edge of a polysome comes into proximity of the membrane, therefore creating conditions in which translated mRNA, if tagged by sRNA, could be cleaved by RNase E. On the other hand, the transcripts for RNase E degradation could encounter the degradosome on the membrane upon their diffusion following the release from the polymerase triggered by the action of Rho helicase. Rho is known to induce transcription termination upon transcription and translation uncoupling that could happen for example due to leading ribosome stalling (61). Alternatively, the *ompD* transcript could undergo the transertion mechanism which was described for *Vibrio parahaemolyticus* (62), and suggested to occur also in *E. coli* (63,64). Transertion, a coupling of transcription, translation and membrane insertion of the membrane component of the type III secretion system, is a process suggested to be common for bacterial membrane proteins, to which OmpD belongs. All the above mechanisms could account for processes of co-translational decay (65,66).

Overall, the observations presented here show that the RNA degradosome makes use of its non-nucleolytic components to initiate sRNA-targeted mRNA degradation on 30S-bound mRNA. The capacity to act on a transcript engaged with the PIC illustrates how mRNA surveillance may be integrated with coupled processes of gene expression. Additional mechanisms that control elongation of transcription and translation, and the kinetic and physical interplay between the involved machineries remain interesting questions for future studies.

## Supplementary Material

gkae455_Supplemental_File

## Data Availability

The data underlying this article are available in the article and in its online supplementary material. Further data supporting the findings of this study are available from the corresponding author on request.
