# Supplementary material for: Cooperation of regulatory RNA and the RNA degradosome in transcript surveillance
Source: Nucleic Acids Res. 2024 Jun 6;52(15):9161–73. doi: 10.1093/nar/gkae455 (PMC11347162; doi:10.1093/nar/gkae455)

Supplementary Information

# Cooperation of regulatory RNA and the RNA degradosome in transcript surveillance

Katarzyna J. Bandyra[1,+,*], Kathrin S. Fröhlich[2,3], Jörg Vogel[2,4], Marina Rodnina[5], Akanksha Goyal[5], Ben F. Luisi[1,*]

[1]Department of Biochemistry, Sanger Building, University of Cambridge, Tennis Court Road, Cambridge, CB2 1GA, UK
[+]Current address: Department of Chemistry, Biological and Chemical Research Centre, University of Warsaw, Zwirki i Wigury 101, 02-089 Warsaw, Poland
[2]Institute for Molecular Infection Biology, University of Würzburg, Josef-Schneider-Str. 2, 97080 Würzburg, Germany
[3]Institute of Microbiology, Friedrich Schiller University Jena, 07743 Jena, Germany
[4]Helmholtz Institute for RNA-based Infection Research (HIRI), Helmholtz Center for Infection Research (HZI), Josef-Schneider-Str. 2, 97080 Würzburg, Germany
[5]Max Planck Institute for Multidisciplinary Sciences, Göttingen, Germany
*Correspondence to: bfl20@cam.ac.uk, k.bandyra@uw.edu.pl

## Supplementary Table

**Supplementary Table 1.** Thermodynamic parameters for the binding of seed regions of MicC and its variants to the complementary target sequence in *ompD* from isothermal titration calorimetry.

| MicC variant | ΔH (kcal/mol) | ΔG | ΔS | Kd (M) | n |
|---|---|---|---|---|---|
| 12mer W1 | -59.47 | -10.51 | -161.5 | $2.15*10^{-8}$ | 1.1 |
| 12mer W2 | -42.18 | -9.59 | -107.5 | $1.46*10^{-7}$ | 1.1 |
| 12mer W3 | -26.40 | -9.60 | -55.4 | $1.19*10^{-7}$ | 1.2 |
| **12mer wt** | **-61.29** | **-10.21** | **-168.5** | **$3.59*10^{-8}$** | **0.9** |
| 12mer S1 | -53.54 | -11.55 | -138.5 | $5.26*10^{-9}$ | 1.0 |
| 12mer S2 | -68.46 | -11.92 | -186.5 | $2.76*10^{-9}$ | 0.9 |
| 12mer S3 | -62.76 | -12.44 | -166.0 | $1.56*10^{-9}$ | 0.9 |

# Supplementary Figures

**Supplementary Figure 1. Degradation of *ompD*$_{118}$-PIC by RNase E.** (A) Quantification of the degradation of 200 nM *ompD*$_{118}$ in the presence of different 200 nM MicC variants (WT and mutants W1, W2, W3, S1, S2, S3) and 200 nM Hfq by 200 nM RNase E (1-529) (left panel) and 50 nM full degradosome (right panel). The same reactions were performed using *ompD*$_{118}$-PIC as a substrate for RNase E (1-529) (B), RNase E (1-850)/RhlB/Enolase (C) and full degradosome (D). The activity of a recombinant degradosome preparation comprising RNase E 1-850, RhlB and enolase, and which is free of PNPase, shows stronger relative activity compared with the isolated catalytic domain of RNase E; however, it is not as efficient as the whole degradosome.

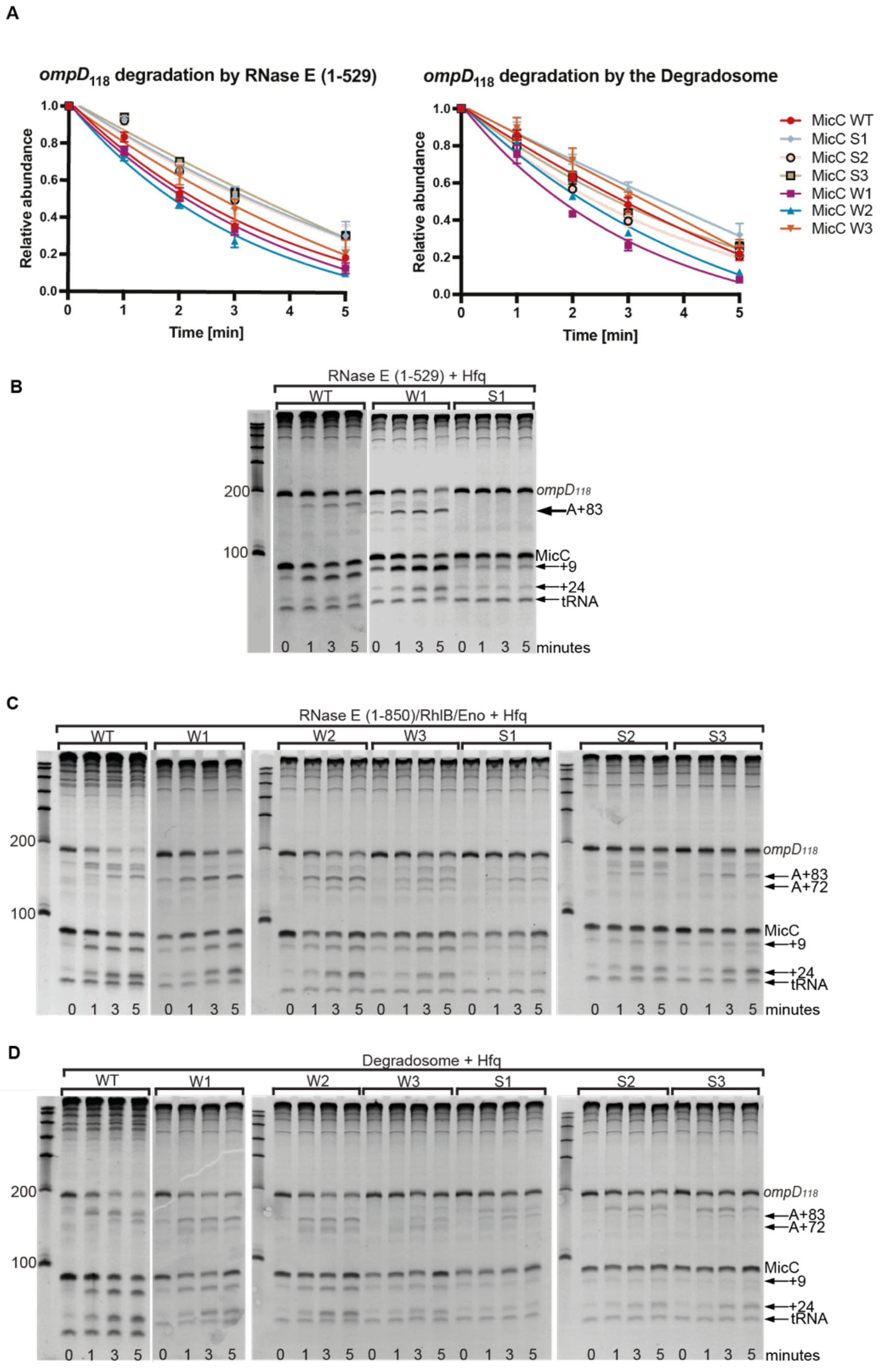

**Supplementary Figure 2.** The influence of RNase III and MicC seed strength on *ompD* degradation *in vivo* (A) and *in vitro* (B). (A) Northern blot for MicC WT and mutants MicC-S3 and MicC-W1 in cell extracts upon expression from an inducible $P_{BAD}$ promoter or a control plasmid in Salmonella strain that is deficient for RNase III (*rnc*). Samples were obtained before and 2, 4, and 8 minutes after addition of L-arabinose. Processed RNA species are indicated by arrows. Probing for 5S rRNA serves as loading control. A plot of the relative abundance of *ompD* full-length species at indicated time-points represents the average result and the standard deviation of three biological replicates. (B) *ompD* degradation by RNase III in the presence and absence of MicC WT and mutants. The same amount of RNA (200 nM) was used in each reaction.

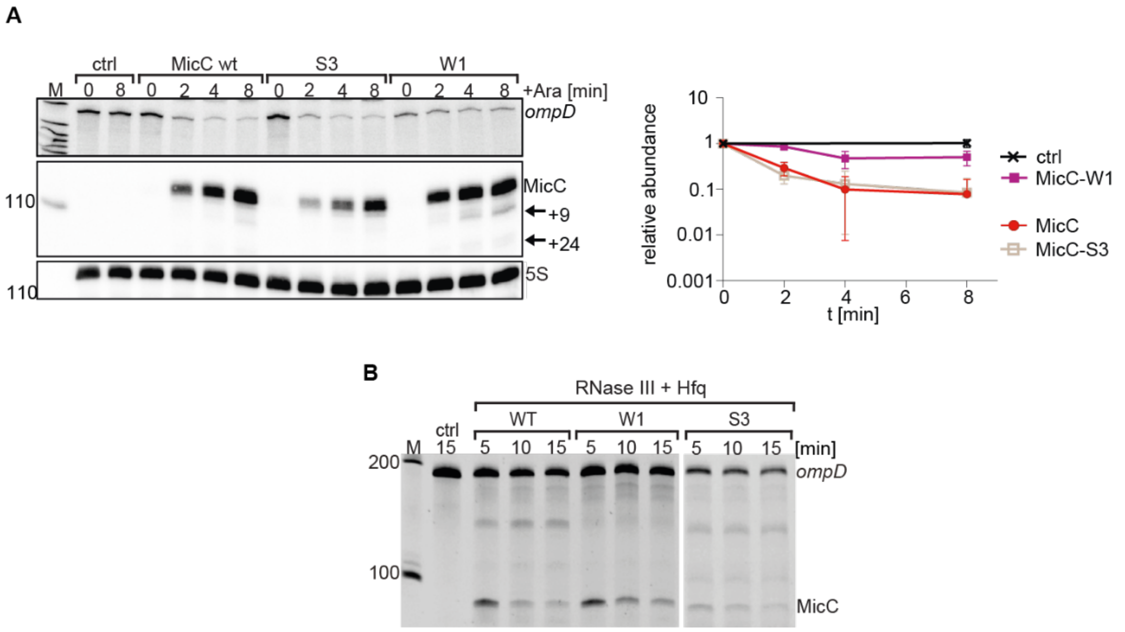

Supplement: gkae455_Supplemental_File [file gkae455_supplemental_file.pdf]
